# Prehabilitation in elective abdominal cancer surgery in older patients: systematic review and meta‐analysis

**DOI:** 10.1002/bjs5.50347

**Published:** 2020-09-22

**Authors:** S. L. Daniels, M. J. Lee, J. George, K. Kerr, S. Moug, T. R. Wilson, S. R. Brown, L. Wyld

**Affiliations:** ^1^ Academic Directorate of General Surgery Sheffield UK; ^2^ Directorate of Anaesthesia, Sheffield Teaching Hospitals NHS Foundation Trust Sheffield UK; ^3^ Department of Oncology and Metabolism, University of Sheffield Sheffield UK; ^4^ Clinical Research Academy, School of Health and Related Research, University of Sheffield Sheffield UK; ^5^ Department of Surgery, Royal Alexandra Hospital Paisley UK; ^6^ Doncaster and Bassetlaw Teaching Hospitals NHS Foundation Trust Doncaster UK

## Abstract

**Background:**

Prehabilitation has emerged as a strategy to prepare patients for elective abdominal cancer surgery with documented improvements in postoperative outcomes. The aim of this study was to assess the evidence for prehabilitation interventions of relevance to the older adult.

**Methods:**

Systematic searches were conducted using MEDLINE, Web of Science, Scopus, CINAHL and PsychINFO. Studies of preoperative intervention (prehabilitation) in patients undergoing abdominal cancer surgery reporting postoperative outcomes were included. Age limits were not set as preliminary searches revealed this would be too restrictive. Articles were screened and selected based on PRISMA guidelines, and assessment of bias was performed. Qualitative, quantitative and meta‐analyses of data were conducted as appropriate.

**Results:**

Thirty‐three studies (3962 patients) were included. Interventions included exercise, nutrition, psychological input, comprehensive geriatric assessment and optimization, smoking cessation and multimodal (two or more interventions). Nine studies purposely selected high‐risk, frail or older patients. Thirty studies were at moderate or high risk of bias. Ten studies individually reported benefits in complication rates, with meta‐analyses for overall complications demonstrating significant benefit: multimodal (risk difference −0·1 (95 per cent c.i. −0·18 to −0·02); *P* = 0·01, *I* ^2^ = 18 per cent) and nutrition (risk difference −0·18 (−0·26 to −0·10); *P* < 0·001, *I* ^2^ = 0 per cent). Seven studies reported reductions in length of hospital stay, with no differences on meta‐analysis.

**Conclusion:**

The conclusions of this review are limited by the quality of the included studies, and the heterogeneity of interventions and outcome measures reported. Exercise, nutritional and multimodal prehabilitation may reduce morbidity after abdominal surgery, but data specific to older patients are sparse.

## Graphical abstract

This systematic review and meta‐analysis suggests that multimodal and nutrition‐alone prehabilitation interventions may reduce postoperative complications and length of hospital stay. More high‐quality research is needed specifically on prehabilitation for older patients.

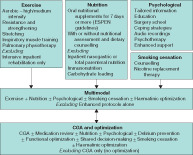

Use multimodal prehabilitation to reduce morbidity

## Abstract (es)

**Antecedentes:**

La pre‐habilitación ha surgido como una estrategia para preparar a los pacientes para la cirugía electiva del cáncer abdominal con mejoras documentadas en los resultados postoperatorios. El objetivo de este estudio fue evaluar la evidencia sobre las intervenciones de pre‐habilitación relevantes en adultos de edad avanzada.

**Métodos:**

Se realizaron búsquedas sistemáticas utilizando MEDLINE, Web of Science, Scopus, CINAHL y PsychINFO. Registro PROSPERO: CRD42019120381. Se incluyeron estudios de intervención preoperatoria (pre‐habilitación) en pacientes sometidos a cirugía oncológica abdominal que describiesen resultados postoperatorios. No se fijaron límites en la edad dado que las búsquedas preliminares revelaron que ello sería demasiado restrictivo. Los artículos fueron examinados y seleccionados en base a las guías PRISMA y se realizó una evaluación del sesgo. Se llevó a cabo un análisis cualitativo, cuantitativo y metaanálisis de los datos según fuese apropiado.

**Resultados:**

Se incluyeron 33 estudios (3.962 patients). Las intervenciones incluyeron ejercicio, nutrición, intervención psicológica, evaluación geriátrica global y optimización, abandono del tabaquismo y multimodal (dos o más intervenciones). Nueve estudios seleccionaron expresamente una población de pacientes de elevado riesgo, frágiles o de edad avanzada. Treinta estudios presentaban un riesgo moderado/alto de sesgo. Diez estudios describieron de forma individual beneficios en las tasas de complicaciones con metaanálisis para las complicaciones globales demostrando un beneficio significativo: multimodal (diferencia de riesgo ‐0,1 (i.c. del 95% −0,18 a −0,02); *P* = 0,01, I^2^ = 18%) e intervención nutricional (diferencia de riesgo −0,18 (i.c. del 95% −0,26 a −0,10); *P* < 0,001, I^2^ = 0%). Siete estudios describieron reducciones en la duración de la estancia hospitalaria, sin diferencias en el metaanálisis.

**Conclusión:**

Las conclusiones de esta revisión están limitadas por la calidad de los estudios incluidos, heterogeneidad de las intervenciones y descripción de las medidas de resultados. Las intervenciones de pre‐habilitación de ejercicio, nutricionales y multimodales puede reducir la morbilidad tras cirugía abdominal, pero los datos concretos en pacientes de edad avanzada son escasos.

## Introduction

The majority of cancers in the UK are diagnosed in the older adult population (aged 65 years and above), with this population predicted to increase exponentially^1^. The pathogenesis and treatment of cancer can lead to a decline in cardiorespiratory fitness, weight loss and psychological morbidity^2^. Surgery remains the mainstay of curative treatment for many gastrointestinal, gynaecological and urological cancers, but outcomes are poorer in the older adult, making strategies to optimize this complex group increasingly important.

**Fig. 1:**
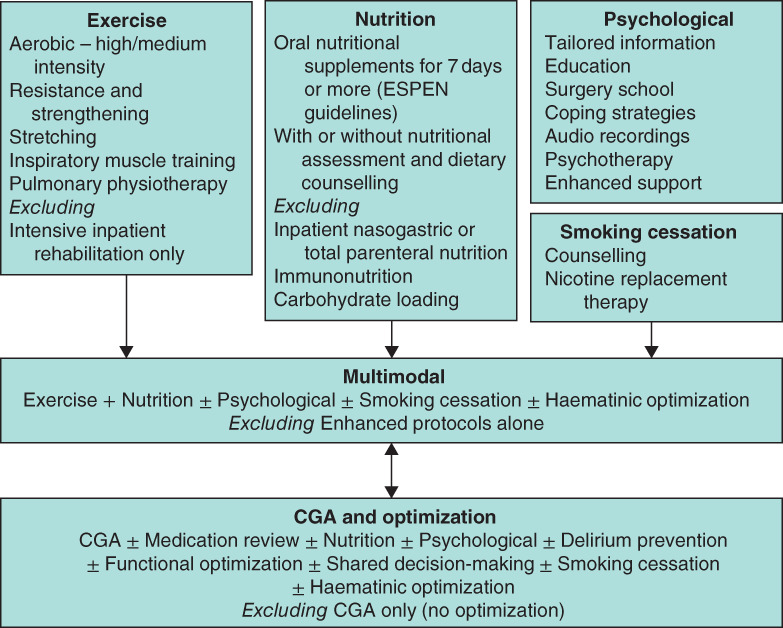
Summary of prehabilitation intervention components and exclusions ESPEN, European Society for Clinical Nutrition and Metabolism; CGA, comprehensive geriatric assessment.

**Fig. 2:**
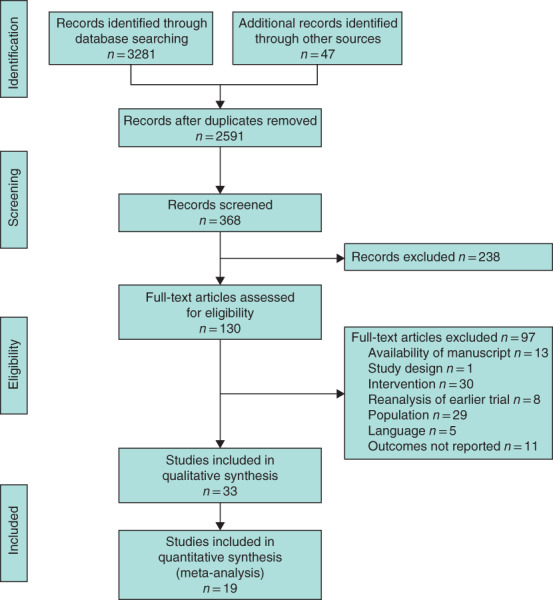
PRISMA diagram for the review

Adverse factors associated with ageing include co‐morbidity, polypharmacy, cognitive impairment, dependency and frailty, all of which are associated with increased all‐cause mortality in the general population^3^. When these at‐risk individuals are exposed to the stress of major cancer surgery, postoperative mortality and morbidity also increase^4^, ^5^. Common lifestyle choices, including smoking, poor nutrition and sedentary behaviours, add to this risk. ‘Prehabilitation’, the process of enhancing an individual's functional capacity before elective surgery with the aim of improving tolerance to the anticipated physiological stress of major surgery, may have a role in improving postoperative outcomes^6^. Prehabilitation programmes vary in their components, but can include exercise programmes, nutritional or psychological interventions^7^. Where they encompass different types of intervention, they are referred to as ‘multimodal’^8^. In the context of the older adult, programmes may also include preoperative comprehensive geriatric assessment (CGA) and optimization. A summary of intervention types is presented in *Fig*. *1*.

Early prehabilitation studies focused on the safety and feasibility of unimodality interventions^9^. More recently, studies have been more likely to be multimodal and to involve higher‐risk populations^10^. Previous systematic reviews^11^, ^12^, ^13^, ^14^, ^15^, ^16^, ^17^, ^18^, ^19^, ^20^ focused predominantly on single‐modality prehabilitation in mixed surgical populations. This review addresses the need for an updated review of the entire spectrum of prehabilitation interventions in elective abdominal cancer surgery with particular relevance to the older patient.

## Methods

This systematic review and meta‐analysis was conducted with reference to the Cochrane Handbook and is reported using the PRISMA guidelines^21^. The protocol was registered with PROSPERO (CRD42019120381). The primary objective was to determine whether any modality of prehabilitation (alone or in combination) before elective abdominal surgery leads to a reduction in either length of hospital stay (LOS) or complications (overall, pulmonary, wound infection rate, delirium, severe complications) compared with a control arm that does not include prehabilitation. The review was undertaken with particular relevance to older adults. Secondary objectives were to determine any effect on functional outcome measures (physical activity or walking capacity, weight loss, discharge independence) and psychological outcome measures (quality of life (QoL)).

### Search strategy

Systematic searches were performed of the MEDLINE, Web of Science, Scopus, Cumulative Index to Nursing and Allied Health Literature (CINAHL), PsychINFO and the Cochrane databases for papers published from database inception to January 2019. Preliminary searches revealed that limiting the searches to studies performed in older adults would be too restrictive and result in the exclusion of potentially relevant studies; therefore no age limits were set. Searches were limited to studies published in the English language as resources were not available to support translation. The search was constructed using the PICO (patient, intervention, comparison, outcome) framework: Patient (adults undergoing abdominal or gastrointestinal surgery); Intervention (prehabilitation or preoperative optimization); Comparator (standard care or rehabilitation only); and Outcome (primary: LOS or complication rates). Clinical.Trials.gov was also searched for trials that had been completed but not published. A sample search strategy is shown in *Appendix* *S1* (supporting information).

### Inclusion and exclusion criteria

Randomized, case–control, cohort or retrospective studies reporting on adults (aged 18 years or above) undergoing surgery with curative intent for any gastrointestinal (oesophagus, stomach, pancreas, liver, colorectal) or intra‐abdominal (urological or gynaecological) cancer were included. Studies including mixed surgical populations were included if they reported the cancer and non‐cancer results separately or if more than 50 per cent of the population were patients with cancer. Studies could test any prehabilitation intervention or preoperative optimization strategy, alone or in combination (multimodal), and had to report outcomes in a control group. Control groups could include standard care, placebo, postoperative rehabilitation programme only, information leaflet or verbal advice on preparing for surgery and positive behaviour change (for example smoking cessation or alcohol reduction) in line with current perioperative care guidelines. Studies of postoperative interventions only were excluded, as were studies that did not report on either of the primary outcomes. Studies published only in abstract form without full text were excluded. Reference lists of primary studies and relevant systematic reviews were also hand‐searched for additional studies.

Screening of all titles and abstracts was undertaken independently by two reviewers. Articles were considered for full‐text review if they met the study inclusion criteria or could not be excluded on the basis of the abstract alone. Full‐text articles were retrieved and assessed by the same two reviewers. Disagreements were addressed by discussion and consensus and, if required the opinion of a third reviewer was sought.

### Definitions of eligible interventions

Eligible interventions included exercise interventions (either alone or in combination with pulmonary exercises), nutritional assessment and supplementation, psychological interventions, CGA and optimization, smoking cessation and multimodal (two or more modalities). These are summarized in *Fig*. *1*.

### Assessment of study quality

Risk‐of‐bias assessment was performed using the Cochrane risk‐of‐bias tool^22^ for randomized trials and the Risk of Bias In Non‐randomized Studies – of Interventions (ROBINS‐I) ^23^ for non‐randomized trials. Randomized studies were graded for risk of bias (+, low risk; −, high risk; ?, unclear risk) in each of the following domains: sequence generation, allocation concealment, blinding, incomplete outcome data, selective outcome reporting and other source of bias. Non‐randomized studies were assessed on bias due to confounding, selection, classification of interventions, deviations from intended interventions, missing data, outcome measurement and reporting. Quality assessment was undertaken independently by two reviewers, and disagreements were resolved by consensus.

### Data extraction

Data were extracted according to a predesigned pro forma, which included study characteristics, baseline data, intervention characteristics, adherence and outcomes. Studies were divided according to modality: exercise (alone or including pulmonary training), multimodal, nutrition, psychological, smoking, and CGA with optimization.

The primary outcomes, LOS and complication rates, were recorded as mean(s.d.) values and proportions respectively. Where the mean was not reported, an approximation was calculated from the median and range^22^. Complication rates were recorded as total, severe (Clavien–Dindo grade III or above) or pulmonary complications, wound infections and delirium within 30 days of surgery. Secondary outcomes were extracted where reported: change in functional outcome measures (preoperative change in 6‐minute walk test (6MWT) or cardiopulmonary exercise test (CPET) variables of physiological fitness, percentage preoperative weight loss or discharge independence), or psychological outcomes (postoperative Hospital Anxiety and Depression Scale (HADS), Short Form 36 Health Survey (SF‐36®; Rand Corporation, Santa Monica, California, USA) or European Organisation for Research and Treatment of Cancer Quality of Life Questionnaire Core 29 and 30 (EORTC QLQ‐C29/C30) score).

**Fig. 3:**
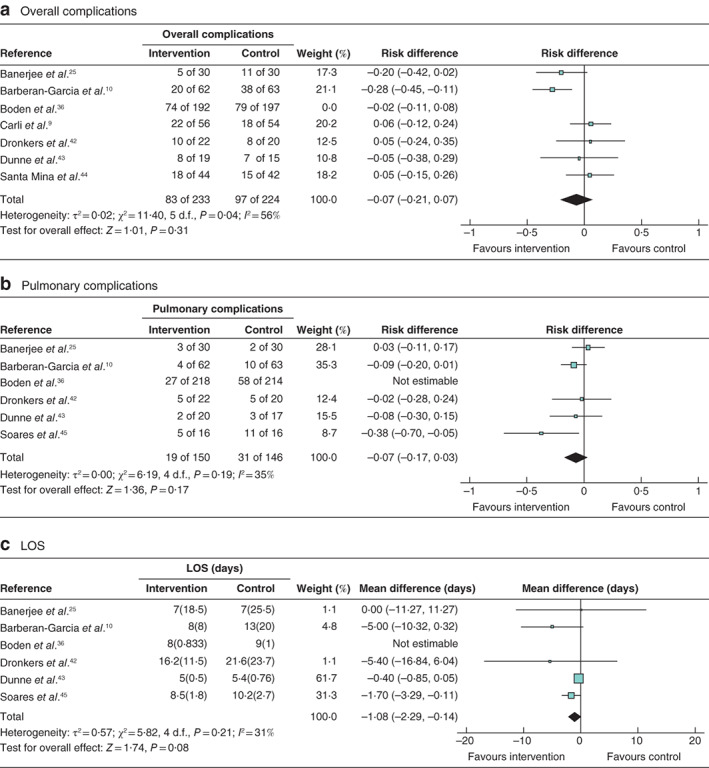
Forest plots showing the effect of exercise prehabilitation on overall and pulmonary complications, and length of hospital stay **a** Overall complications; **b** pulmonary complications; **c** mean(s.d.) length of hospital stay (LOS). **a,b** Mantel–Haenszel random‐effects models were used for meta‐analysis; risk differences are shown with 95 per cent confidence intervals. **c** An inverse‐variance model was used for meta‐analysis; mean differences are shown with 95 per cent confidence intervals.

**Fig. 4:**
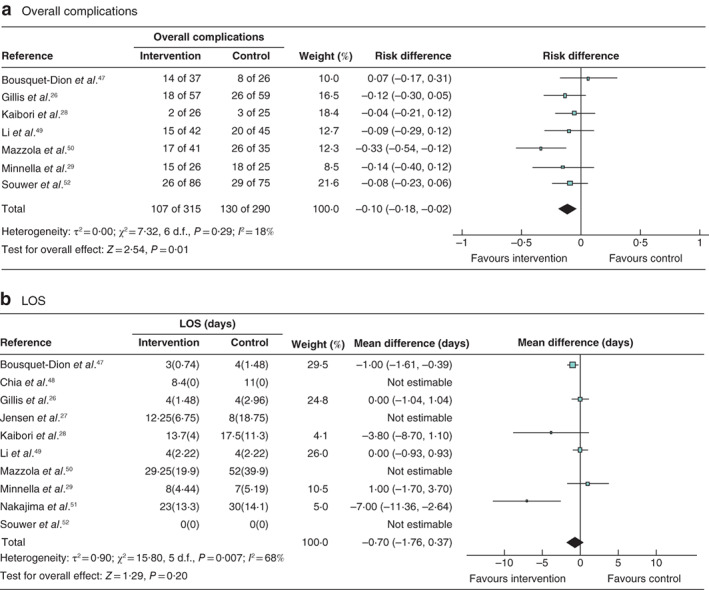
Forest plots showing the effect of multimodal prehabilitation on overall complications and length of hospital stay **a** Overall complications; **b** mean(s.d.) length of hospital stay (LOS). **a** A Mantel–Haenszel random‐effects model was used for meta‐analysis; risk differences are shown with 95 per cent confidence intervals. **b** An inverse‐variance model was used for meta‐analysis; mean differences are shown with 95 per cent confidence intervals.

### Statistical analysis

Qualitative analyses were performed for all studies that met the inclusion criteria. Studies were analysed according to the type of prehabilitation intervention. Meta‐analysis was performed using RevMan software (Review Manager version 5.3, 2014; The Cochrane Collaboration, The Nordic Cochrane Centre, Copenhagen, Denmark) where the number (greater than 3) and quality of studies permitted, if the 95 per cent c.i. overlapped and effect sizes were similar^24^. Meta‐analysis was performed using random‐effects models, assessing risk difference for both dichotomous and continuous outcomes. Heterogeneity was assessed using the *I* ^2^ statistic. Significance was set at α = 0·050.

**Fig. 5:**
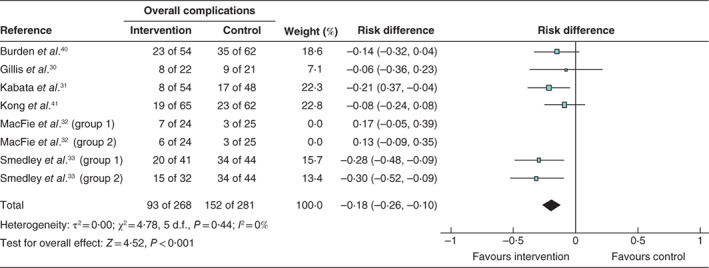
Forest plot showing the effect of nutrition prehabilitation on overall complications A Mantel–Haenszel random‐effects model was used for meta‐analysis; risk differences are shown with 95 per cent confidence intervals.

## Results

Searches were performed on 6 January 2019. Some 130 papers were identified for full text review; 97 were excluded, leaving 33 studies for inclusion (*Fig*. *2*). There were 25 RCTs (including pilot and feasibility studies)^9^, ^10^, ^25^, ^26^, ^27^, ^28^, ^29^, ^30^, ^31^, ^32^, ^33^, ^34^, ^35^, ^36^, ^37^, ^38^, ^39^, ^40^, ^41^, ^42^, ^43^, ^44^, ^45^, ^46^, ^47^, seven prospective cohort studies (with either contemporary or historical controls)^48^, ^49^, ^50^, ^51^, ^52^, ^53^, ^54^, and one retrospective study^55^. Three studies^32^, ^33^, ^35^ reported two separate intervention groups, resulting in a total of 36 interventions for comparison (*Table S1*, supporting information).

### Baseline characteristics

The studies, published between 2000 and 2019, included 2028 patients undergoing prehabilitation and 1934 controls. Interventions comprised: exercise only (9 studies)^9^, ^10^, ^25^, ^36^, ^42^, ^43^, ^44^, ^45^, ^46^, multimodal (10 studies)^26–29,47–52^, nutrition only (7 studies)^30^, ^31^, ^32^, ^33^, ^40^, ^41^, ^53^, psychological only (2 studies)^34^, ^35^, CGA with optimization only (4)^37^, ^38^, ^54^, ^55^ and smoking cessation only (1 study)^39^. Sample sizes ranged from 32 to 443 patients, with most having fewer than 100 patients in each arm; only four studies^36^, ^37^, ^54^, ^55^ had more than this, and were mostly non‐randomized. The wide range of sample sizes reflects the diverse primary outcomes on which power calculations were based, and also the fact that a small number were pilot or feasibility studies. Studies were predominantly single‐centre, with only eight studies^33^, ^36^, ^37^, ^38^, ^40^, ^44^, ^45^, ^53^ conducted across multiple centres. Studies were conducted in North America, Europe, Australasia, South‐East Asia and Brazil. A range of surgical populations were studied, including colorectal (16 studies), upper gastrointestinal, hepatobiliary and pancreatic (9 studies), urological (3 studies), and mixed populations of gastrointestinal and abdominal malignancies (5 studies) (*Table S1*, supporting information).

Twenty‐four studies involved patients with cancer exclusively, with a range of 52–78 per cent of patients with cancer in the remaining studies. Six studies included patients receiving neoadjuvant therapy. Although the average age range was 55–81 years, it was less than 70 years in the majority of studies. Three^48^, ^50^, ^52^ of the ten multimodal studies and four^37^, ^38^, ^54^, ^55^ of the CGA studies had populations with an average age over 75 years (*Table S1*, supporting information). Nine studies^10^, ^37^, ^38^, ^42^, ^48^, ^50^, ^52^, ^54^, ^55^ selected patients who were either assessed as frail (using a recognized frailty screen or criteria) or over a certain age cut‐off; however the method of detecting frailty, frailty criteria used, and age varied between studies. Two studies^40^, ^41^ selected patients who were malnourished, and one^28^ selected patients with chronic liver injury (*Table S1*, supporting information).

### Methodological quality assessment

The assessment of methodological quality is summarized in *Tables* *1* and *2*. Only three randomized studies blinded both participants and researchers, one^30^ by using a placebo oral nutritional supplement, the second^36^ by having all patients attend a preoperative physiotherapy appointment in which those in the control arm received only an information booklet whereas patients in the intervention arm learned breathing exercises, and the third^10^ by using a double‐informed consent model where control and intervention arms were not aware of each other. The absence of blinding of either participants or study personnel was the most common reason for high risk of bias assessment. The majority of RCTs adequately described randomization, but allocation concealment was not as reported robustly. Half of the RCTs adequately described blinding of outcome assessment^10,25,26,29,30,34–36,38–40,42,43^. Only two studies^27^, ^34^ did not adequately report their outcome data (*Table* *1*).

**Table 1:** Cochrane risk‐of‐bias tool results for randomized studies

Reference	Randomization (selection bias)	Allocation concealment (selection bias)	Blinding of participants and personnel (performance bias)	Blinding of outcome assessment (detection bias)	Incomplete outcome data (attrition bias)	Selective reporting (reporting bias)	Other sources of bias (other bias)
**Exercise alone**							
Banerjee *et al*.^25^	+	+	−	+	+	?	?
Barberan‐Garcia *et al*.^10^	+	+	+	+	+	+	?
Boden *et al*.^36^	+	+	+	+	+	?	?
Carli *et al*.^9^	+	?	−	?	+	?	?
Dronkers *et al*.^42^	+	?	−	+	+	?	?
Dunne *et al*.^43^	+	+	−	+	+	?	?
Santa Mina *et al*.^44^	?	?	−	?	+	+	?
Soares *et al*.^45^	?	?	−	−	+	+	?
Yamana *et al*.^46^	?	?	−	−	+	?	?
**Multimodal**							
Bousquet‐Dion *et al*.^47^	+	+	−	−	+	?	?
Gillis *et al*.^26^	+	+	−	+	+	?	?
Jensen *et al*.^27^	+	+	−	−	?	+	?
Kaibori *et al*.^28^	?	?	−	?	+	?	?
Minnella *et al*.^29^	+	+	−	+	+	+	?
**Nutrition**							
Burden *et al*.^40^	+	+	−	+	+	+	?
Gillis *et al*.^30^	+	+	+	+	+	?	?
Kabata *et al*.^31^	+	+	−	?	+	?	?
Kong *et al*.^41^	+	?	−	−	+	?	?
MacFie *et al*.^32^	?	?	−	?	+	?	?
Smedley *et al*.^33^	?	?	−	?	+	?	?
**Psychological**							
Chaudhri *et al*.^34^	?	?	−	+	?	?	?
Haase *et al*.^35^	?	?	−	+	+	?	?
**CGA and optimization**							
Hempenius *et al*.^37^	+	+	−	?	+	?	?
Ommundsen *et al*.^38^	+	+	−	+	+	?	?
**Smoking**							
Sørensen and Jørgensen^39^	+	+	−	+	+	?	?

+, Low risk of bias; −, high risk of bias; ?, unclear risk of bias. CGA, comprehensive geriatric assessment.

**Table 2:** ROBINS‐I tool results for non‐randomized studies

Reference	Type of study	Bias due to confounding	Bias in selection of participants	Bias in classification of interventions	Bias due to deviations from intended interventions	Bias due to missing data	Bias in measurement of outcomes	Bias in selection of reported result
**Multimodal**								
Chia *et al*.^48^	Prospective, before and after intervention	Moderate	High	Low	Low	Low	Moderate	Low
Li *et al*.^49^	Prospective, before and after intervention	Moderate	Low	Low	Low	Low	Moderate	Low
Mazzola *et al*.^50^	Prospective cohort, retrospective control	Moderate	Low	Low	Low	Low	Moderate	Low
Nakajima *et al*.^51^	Prospective cohort, retrospective control	Moderate	Moderate	Low	Low	Low	Moderate	Low
Souwer *et al*.^52^	Prospective, before and after intervention	Moderate	Low	Low	Low	Low	Moderate	Low
**Nutrition**								
Maňásek *et al*.^53^	Prospective cohort, retrospective control	Moderate	Moderate	Low	Low	Low	Moderate	Low
**CGA and optimization**								
Indrakusuma *et al*.^55^	Retrospective cohort	Moderate	Moderate	Moderate	Low	Low	Moderate	Low
McDonald *et al*.^54^	Case–control (matched)	Moderate	Low	Low	Low	Low	Moderate	Low

CGA, comprehensive geriatric assessment.

Seven^49^, ^50^, ^51^, ^52^, ^53^, ^54^, ^55^ of the eight non‐randomized studies were graded as moderate risk of bias owing to bias in outcome measurements and due to confounding factors as they mainly used historical controls. One study^48^ was judged to be at high risk of bias as the authors chose to include a wider age range in the intervention group than in controls (*Table* *2*).

### Interventions

#### Exercise‐based interventions

Unimodal exercise interventions were most commonly based in hospital and conducted under supervision^36^, ^42^, ^43^, ^45^, ^46^; four studies^36^, ^42^, ^45^, ^46^ included specific pulmonary exercises or training. Exercise prehabilitation programmes varied in intensity from a single preoperative session^36^ to one to three times per week, and ranged from 1 to 6 weeks in duration.

#### Multimodal interventions

Multimodal interventions were more likely to be home‐based^26^, ^29^, ^49^, ^50^, ^51^; all included exercise and nutrition, with four^26^, ^47^, ^49^, ^52^ also including psychological interventions. The nutritional component of multimodal interventions commonly involved dietician assessment and supplementation if required. Two studies^28^, ^48^ did not mention supplementation. Two multimodal programmes specifically mentioned other behavioural modifications: alcohol reduction^49^ and smoking cessation^50^.

#### Nutrition‐based interventions

All nutrition‐only prehabilitation studies^30^, ^31^, ^32^, ^33^, ^40^, ^41^, ^53^ included oral nutritional supplementation, but the prescriptions varied from ‘ad libitum’ between meals to 400 ml three times a day, with duration varying from 1 to 4 weeks. Two studies^32^, ^33^ included separate intervention groups that received supplements both before and after surgery.

#### Psychology‐based interventions

The two psychological prehabilitation studies had different interventions; the study by Chaudhri and colleagues^34^ looked at the impact of a community‐based stoma education intervention, whereas that by Haase and colleagues^35^ involved giving patients audio recordings with either guided imagery or relaxation techniques to listen to before surgery.

#### Comprehensive geriatric assessment with optimization

All four CGA prehabilitation studies^37^, ^38^, ^54^, ^55^ involved preoperative CGA performed by a geriatrician‐led multidisciplinary team, nutritional optimization and medication reviews; two studies^37^, ^54^ included postoperative daily reviews by a geriatric specialist nurse. Two studies specified that they corrected anaemia with either blood transfusion^55^ or supplementation^38^.

#### Smoking cessation

One study^39^ of a smoking cessation intervention met the inclusion criteria; the intervention involved a single smoking cessation counselling session combined with nicotine replacement therapy.

### Adherence

Adherence was reported in eight^9,10,25,36,42–44,46^ of the nine studies of exercise, five^26^, ^27^, ^29^, ^47^, ^49^ of the ten multimodal studies, and four^30^, ^32^, ^40^, ^41^ of the seven nutrition prehabilitation studies, with percentages varying from 69 to 100 per cent, 59 to 98 per cent, and 75 to 99 per cent respectively. Adherence was not stated in studies of psychological, CGA with optimization, or smoking cessation interventions; as these were typically single preoperative interventions, adherence would not have been an issue.

### Primary outcome

Twenty different primary outcomes were reported, and 12 of the 33 studies reported more than one primary outcome measure (*Tables* *3* *–8*). Four studies^25^, ^27^, ^42^, ^44^ reported feasibility as the primary outcome. Postoperative complications (overall complication rate, severe complications (Clavien–Dindo grade II or above, or III or above), pulmonary complications, delirium or site‐specific infection rate) were the most common postoperative outcome measures, and were reported in all except one study^34^. LOS was reported in all except two studies^31^, ^46^.

**Table 3:** Summary of outcomes and results for exercise prehabilitation

**Reference**	**Adherence (%)**	**Primary study outcome**	**Postoperative outcomes** *	**Functional outcomes** *	**Psychological outcomes** *
Banerjee *et al*.^25^	92	Feasibility	All complications: 4 of 30 *versus* 10 of 30, *P* = 0·075 CDC grade ≥ III: 1 of 30 *versus* 4 of 30 Pneumonia: 3 of 30 *versus* 2 of 30 LOS: median 7 (4–78) *versus* 7 (5–107) days	Peak OP: +1·36 (95% c.i. 0·63, 2·10) ml/beat, *P* = 0·001 Peak VE: +7·49 (95% c.i. 2·86, 12·12) l/min. *P* = 0·02 Peak power output: +19 (95% c.i. 10, 27) W. *P* < 0·001	
Barberan‐Garcia *et al*.^10^	87	Any complications	All complications: 20 of 62 *versus* 38 of 63, *P* = 0·001; RR 0·5 (95% c.i. 0·3, 0·8) Pulmonary: 4 of 63 *versus* 10 of 62, *P* = 0·155 Wound: 1 of 63 *versus* 1 of 62 LOS: mean(s.d.): 8(8) *versus* 13(20) days, *P* = 0·078	6MWT: no difference	SF‐36®: PCS n.s. HADS anxiety and depression: no change in either group
Boden *et al*.^36^	98	Pulmonary complications within 14 days	Any complication within 6 weeks: 74 of 192 *versus* 79 of 197 Pulmonary: 27 of 218 *versus* 58 of 214 (adjusted HR 0·48, 95% c.i. 0·30, 0·75, *P* = 0·001) Wound: 36 of 192 *versus* 40 of 197 LOS: median 8 (6–11) *versus* 9 (7–13) days		
Carli *et al*.^9^	79	Change in 6MWT before and after surgery	All complications: 22 of 56 *versus* 18 of 54 CDC grade ≥ III: 6 of 56 *versus* 3 of 54 LOS: mean(s.e.) 11·9(34·6) *versus* 6·6(3·6) days	6MWT: baseline to preop. −10·6(7·3) *versus* + 8·7(6·8) Mean peak *V* o_2_: +134 *versus* + 112 ml/min	HADS anxiety: baseline to postop. follow‐up −1·8(0·7) *versus* −2·0(0·5), *P* n.s. HADS depression: −0·8(0·6) *versus* −0·4(0·5), *P* n.s.
Dronkers *et al*.^42^	97	Feasibility	All complications: 9 of 22 *versus* 8 of 20 Pulmonary: 5 of 22 *versus* 5 of 20 LOS: mean(s.d.) 16·2(11·5) *versus* 21·6 (23·7) days		EORTC QLQ‐C30: *P* n.s.
Dunne *et al*.^43^	92	Oxygen uptake at AT	All complications: 8 of 19 *versus* 7 of 15 CDC grade ≥ III: 3 of 19 *versus* 1 of 15 Pneumonia: 2 of 20 *versus* 3 of 17 Wound: 3 of 20 *versus* 0 of 17 LOS: median (range) 5 (4–6) *versus* 5 (4·5–7) days	*V* o_2_ at AT: +1·5 (95% c.i. 0·2, 2·9) ml per kg per min, *P* = 0·023 Peak work rate: +13 (95% c.i. 4, 22) W, *P* = 0·005	SF‐36 ® overall QoL score: +11 (95% c.i. 1, 21), *P* = 0·028 SF‐36 ® overall mental health score: +11 (1, 22), *P* = 0·037
Santa Mina *et al*.^44^	69	Feasibility	All complications: 18 of 44 *versus* 14 of 42 CDC grade ≥ III: 1 of 44 *versus* 1 of 42 LOS: mean(s.d.) 1·7(0·9) *versus* 1·76(1·0)	6MWT preop.: +14·6(+14·5) (95% c.i. −13·87, 43·05), *P* = 0·313	HADS anxiety postop.: difference estimate +0·47(0·68), *P* = 0·49
Soares *et al*.^45^		Pulmonary function change and 6MWT	Pulmonary: 5 of 16 *versus* 11 of 16, *P* = 0·03 LOS: median (range) 8·5 (4·8–12·3) *versus* 8·5 (6·5–17·3) days	6MWT preop: 514·4 (460–557·5) *versus* 441·5 (412·3–505·9), *P* = 0·105	
Yamana *et al*.^46^	100	Pulmonary complications	Pulmonary (CDC grade ≥ III): 3 of 30 *versus* 5 of 30, *P* = 0·014		

* Comparative data show intervention and control results respectively. CDC, Clavien–Dindo classification; LOS, length of hospital stay; OP, oxygen pulse; VE, minute ventilation; RR, relative risk; 6MWT, 6‐minute walk test; SF‐36®, Short Form 36; PCS, physical component score; HADS, Hospital Anxiety and Depression Scale; HR, hazard ratio; *V* o_2_, oxygen consumption; n.s., not significant; EORTC QLQ, European Organisation for Research and Treatment of Cancer Quality of Life Questionnaire; AT, anaerobic threshold; QoL, quality of life.

### Postoperative, functional and psychological outcomes

#### Exercise studies

One study^10^ reported a significant reduction in overall complications in the intervention arm (20 of 62 *versus* 38 of 63 in the control arm, *P* = 0·001; relative risk 0·5 m, 95 per cent c.i. 0·3 to 0·8). One study^9^ found a non‐significant higher overall complication rate in the intervention arm (22 of 56 *versus* 18 of 54 for the control; *P* value not reported), which was attributed to poor compliance in the intervention group and an increase in physical activity in the control group. Meta‐analysis showed no significant difference in overall complications, but heterogeneity was high (*Fig*. *3a*).

Two studies reported lower rates of pulmonary complications in the intervention group: 27 of 218 *versus* 58 of 214 (adjusted hazard ratio 0·48, 95 per cent c.i. 0·30 to 0·75; *P* = 0·001) in the study by Boden and colleagues^36^, and five of 16 *versus* 11 of 16 (*P* = 0·03) in that of Soares and co‐workers^45^. Yamana *et al*.^46^ also found a lower Clavien–Dindo grade of pulmonary complication with intervention (*P* = 0·014). Meta‐analysis of five studies (the study by Boden and colleagues^36^ was excluded owing to a significantly different intervention) for pulmonary complications revealed a non‐significant trend in favour of the intervention (*Fig*. *3b*).

A non‐significant trend towards lower LOS was also observed on meta‐analysis (*Fig*. *3c* and *Table* *3*).

Two studies^25^, ^43^ that assessed preoperative change in CPET variables before and after intervention both demonstrated significant improvements in peak oxygen uptake and peak work rate (*Table* *3*). Four studies^9^, ^10^, ^44^, ^45^ that assessed functional walking ability using the 6MWT demonstrated no preoperative differences between intervention and control groups. Of the five studies that reported psychological outcomes, only that by Dunne and colleagues^43^ showed an improvement in overall QoL score measured using the SF‐36® (+11, 95 per cent c.i. 1 to 21; *P* = 0·028) and overall mental health score (+11, 1 to 22; *P* = 0·037) (*Table* *3*).

#### Multimodal studies

One study^50^ found a reduction in overall complications in the intervention group (17 of 41 *versus* 26 of 35 in the control group; *P* = 0·005) (*Table* *4*). Meta‐analysis showed a significant reduction in overall complications after multimodal prehabilitation (*Fig*. *4a*). Mazzola and colleagues^50^ (Clavien–Dindo grade II or above: 7 of 41 *versus* 15 of 35 respectively, *P* = 0·02) and Souwer and colleagues^52^ (Clavien–Dindo grade III or above: 14 of 86 *versus* 24 of 75 respectively; odds ratio (OR) 0·4, 95 per cent c.i. 0·2 to 0·9, *P* = 0·03) both showed a reduction in severe complications with multimodal prehabilitation. No other studies demonstrated a reduction in severe complications, delirium, pulmonary or wound infection.

**Table 4:** Summary of outcomes and results for multimodal prehabilitation

**Reference**	**Adherence (%)**	**Primary study** **outcome**	**Postoperative outcomes** *	**Functional outcomes** *	**Psychological outcomes** *
Bousquet‐Dion *et al*.^47^	98	Exercise capacity 6MWT	All complications: 14 of 37 *versus* 8 of 26 Wound: 5 of 37 *versus* 3 of 26 CDC grade ≥ II: 5 of 37 *versus* 4 of 26 CDC grade ≥ III: 2 of 41 *versus* 0 of 39 LOS: median (i.q.r.) 3 (3–4) *versus* 3 (2–4) days, *P* = 0·122	6MWT: mean(s.d.) difference +21(47) *versus* +10(30) m, *P* n.s.	HADS anxiety score > 7: 35% *versus* 23% HADS depression score > 7: 11% *versus* 19%
Chia *et al*.^48^		LOS, complications	Complications (CDC grade ≥ III): 3 of 57 *versus* 5 of 60, *P* = 0·511 LOS: 8·4 *versus* 11 days, *P* = 0·029		
Gillis *et al*.^26^	78	6MWT at 8 weeks	All complications: 12 of 38 *versus* 17 of 39, *P* = 0·277 Wound: 3 of 38 *versus* 3 of 39 CDC grade ≥ III: 4 of 38 *versus* 6 of 39 Pulmonary: 1 of 38 *versus* 0 of 39 LOS: 4 (i.q.r. 3–5) *versus* 4 (3–7) days, *P* = 0·812	6MWT preop.: mean(s.d.) +25·2(50·2) *versus* −16·4(46) m; mean difference 41·7 (95% c.i. 19·8, 63·6) m; adjusted *P* < 0·001	SF‐36®/HADS: *P* n.s.
Jensen *et al*.^27^	59	Feasibility	All complications: 30 of 50 *versus* 34 of 57 LOS: median 8 (3–30) *versus* 8 (4–55), *P* = 0·68		
Kaibori *et al*.^28^		Whole body mass and fat mass	All complications: 2 of 23 *versus* 3 of 23, *P* = 0·671 LOS: mean(s.d.) 13·7(4·0) *versus* 17·5(11·3), *P* = 0·12		
Li *et al*.^49^	70 (partial)	6MWT at 8 weeks	All complications: 15 of 42 *versus* 20 of 45 CDC grade ≥ III: 2 of 42 *versus* 1 of 45 LOS: median (i.q.r.) 4 (3–6) *versus* 4 (3–6) days	6MWT preop.: 464(92) *versus* 402(57) m baseline (prehabilitation group only), *P* < 0·01	SF‐36®: *P* n.s.
Mazzola *et al*.^50^		Mortality, complications	All complications: 17 of 41 *versus* 26 of 35, *P* = 0·005 CDC grade ≥ III: 7 of 41 *versus* 15 of 35, *P* = 0·02 Pulmonary: 2 of 41 *versus* 1 of 35 LOS: median (range) 17 (7–76) *versus* 27 (8–146) days, *P* = 0·08		
Minnella *et al*.^29^	63	6MWT before and after surgery	All complications: 14 of 24 *versus* 18 of 25 CDC grade ≥ II: 12 of 24 *versus* 16 of 25 CDC grade ≥ III: 6 of 24 *versus* 10 of 25 LOS: median (i.q.r.) 8 (5·75–11·75) *versus* 7 (5·5–12·5) days, *P* = 0·44	6MWT preop.: mean(s.d.) change +36·9(51·4) *versus* −22·8(52·5) m, *P* < 0·001	
Nakajima *et al*.^51^		Preop. nutritional status and postop. course	Complications (CDC grade ≥ III): 32 of 76 *versus* 38 of 76 Pneumonia: 1 of 76 *versus* 1 of 76 Wound: 2 of 76 *versus* 3 of 76 LOS: median (i.q.r.) 23 (16–34) *versus* 30 (21–40) days, *P* = 0·045	Prehabilitation (no control) 6MWT: median (i.q.r.) baseline 530 (470–571) to preop. 554 (499–620) m, *P* < 0·001	
Souwer *et al*.^52^		1‐year mortality	All complications: 24 of 86 *versus* 26 of 63 CDC grade ≥ III: 14 of 86 *versus* 24 of 75 (OR 0·4 (95% c.i. 0·2, 0·9), *P* = 0·03) Pulmonary: *P* = 0·3 LOS ≥ 14 days: 5 of 86 *versus* 17 of 63 days (OR 0·2 (0·1, 0·5), *P* = 0·001		

* Comparative data show intervention and control results respectively. 6MWT, 6‐minute walk test; CDC, Clavien–Dindo classification; LOS, length of hospital stay; n.s., not significant; HADS; Hospital Anxiety and Depression Scale; SF‐36®, Short Form 36; OR, odds ratio.

Three studies reported a significant reduction in LOS in the intervention group: 8·4 *versus* 11 days in the control group (*P* = 0·029) in the study by Chia and colleagues^48^; median LOS 23 (i.q.r. 16–34) *versus* 30 (21–40) days in the control group (*P* = 0·045) in the study by Nakajima and co‐workers^51^; and LOS of 14 days or more in five of 86 *versus* 17 of 63 patients respectively (OR 0·2, 95 per cent c.i. 0·1 to 0·5; *P* = 0·001) in the study by Souwer and colleagues^52^ (*Table* *4*). Meta‐analysis for LOS including six studies was not significant; however, there were high levels of heterogeneity (*Fig*. *4b*).

Four multimodal studies^26^, ^29^, ^49^, ^51^ demonstrated significant preoperative improvements in functional walking ability using the 6MWT after the intervention (mean difference range 24–62 m; all *P* < 0·010) (*Table* *4*). However, in two of these studies^49^, ^51^ walking ability was tested only in the intervention group. No differences in psychological outcomes were observed in multimodal studies^47^, ^49^, ^59^ (*Table* *4*).

#### Nutrition studies

Two studies reported a reduction in overall complications in the intervention group: eight of 54 *versus* 17 of 48 in the control group (*P* = 0·04) in the study by Kabata and colleagues^31^, and 15 of 32 *versus* 34 of 44 respectively (*P* < 0·050) for group 2 in the study by Smedley *et al*.^33^ (*Table* *5*). Meta‐analysis demonstrated significantly fewer overall complications following the intervention (the historical study of MacFie *et al*.^32^ was excluded from meta‐analysis) (*Fig*. *5*).

**Table 5:** Summary of outcomes and results for nutrition prehabilitation

Reference	Adherence (%)	Primary study outcome	Postoperative outcomes*	Functional outcomes*	Psychological outcomes*
**Burden *et al*.** ^40^	75 (estimated)	SSI or chest infection	All complications: 23 of 54 *versus* 35 of 62, *P* = 0·114 Pneumonia: 5 of 54 *versus* 4 of 62 CDC grade ≥ III: 9 of 54 *versus* 10 of 62 SSI: 11 of 55 *versus* 17 of 45 (OR 0·41 (95% c.i. 0·16, 1·00), *P* = 0·044) LOS: median (i.q.r.) 7 (4–10·5) *versus* 7 (4–10) days, *P* = 0·63	% weight loss preop.: median (i.q.r.) 4·1 (1·7–7·0) *versus* 6·7 (2·6–10·8), *P* = 0·016	
**Gillis *et al*.** ^30^	93·7–96·6	6MWT before and after surgery	All complications: 8 of 22 *versus* 9 of 21 CDC grade ≥ III: 2 of 22 *versus* 2 of 21 Pneumonia: 0 of 22 *versus* 1 of 21 LOS: median 5 (3–13) *versus* 4 (3–10) days	6MWT: mean(s.d.) +20·8(42·6) *versus* +1·2(65·5) m, *P* = 0·27	SF‐36 ® postop.: PCS 41·3 (34·2–46·5) *versus* 36·5 (34·5–42·8); MCS 47·7 (38·1–53·8) *versus* 41·3 (35·6–55·8)
**Kabata *et al*.** ^31^	–	Complications within 30 days	All complications: 8 of 54 *versus* 17 of 48, *P* = 0·04 CDC grade ≥ III: 5 of 54 *versus* 11 of 48, *P* < 0·001 Wound: 1 of 54 *versus* 7 of 48 Pneumonia: 1 of 54 *versus* 0 of 48	% weight loss preop.: median 7·4 *versus* 6·3, *P* n.s.	
**Kong *et al*.** ^41^	99 (partial)	Postop. complications, CDC grade ≥ II	Complications (CDC grade ≥ III): 9 of 65 *versus* 12 of 62 Wound: 7 of 65 *versus* 3 of 62 Pulmonary: 6 of 65 *versus* 4 of 62 LOS: mean(s.d.) 9·3(3·6) *versus* 9·7(5·9) days	% bodyweight change preop.: −0·37 *versus* −0·97, *P* = 0·173	EORTC‐QLQ: no difference
**MacFie *et al*.** ^32^		Weight change and clinical outcomes		Weight loss preop.: *P* n.s.	
Group 1	89·3		All complications: 7 of 24 *versus* 3 of 25 LOS: mean 12 *versus* 13 days		HADS postop.: anxiety or depression, *P* n.s.
Group 2	80·7		All complications: 6 of 24 *versus* 3 of 25 LOS: mean 11 *versus* 13 days		HADS postop.: anxiety or depression, *P* n.s.
**Maňásek *et al*.** ^53^		Complications	Wound: 3 of 52 *versus* 13 of 105 (RR 2·2) LOS: mean(s.d.) 9·4(5·0) *versus* 12·0(6·4) days, *P* = 0·002	% weight loss postop.: 2·6 *versus* 6·4, *P* n.s.	
**Smedley *et al*.** ^33^		Postop. change in bodyweight			
Group 1	–		All complications: 20 of 41 *versus* 34 of 44 Buzby definition^56^: minor 17 of 41 *versus* 30 of 44; major 3 of 41 *versus* 4 of 44 LOS: mean(s.d.) 12·8(4·5) *versus* 14·1(6·6) days	–	SF‐36®: no difference
Group 2	–		All complications: 15 of 32 *versus* 34 of 44, *P* < 0·05 Buzby definition^56^: minor 10 of 32 *versus* 30 of 44; major 5 of 32 *versus* 4 of 44 LOS: mean(s.d.) 11·7(5·1) *versus* 14·1(6·6) days	Only group to gain weight before surgery; lost less weight over course of study, *P* = 0·05	SF‐36®: no difference

* Comparative data show intervention and control results respectively. SSI, surgical‐site infection; CDC, Clavien–Dindo classification; OR, odds ratio; LOS, length of hospital stay; 6MWT, 6‐minute walk test; SF‐36®, Short Form 36; PCS, physical component score; MCS, mental component score; n.s., not significant; EORTC QLQ, European Organisation for Research and Treatment of Cancer Quality of Life Questionnaire; HADS; Hospital Anxiety and Depression Scale; RR, relative risk.

Kabata and colleagues^31^ also reported a reduction in severe complications in the intervention group (Clavien–Dindo grade III or above: 5 of 54 *versus* 11 of 48 in the control group; *P* < 0·001) and Burden and co‐workers^40^ found a reduction in surgical‐site infection (11 of 55 *versus* 17 of 45; OR 0·41, 95 per cent c.i. 0·16 to 1·00, *P* = 0·044) (*Table* *5*). Only one study^53^ reported a reduction in LOS with the intervention (mean(s.d.) 9·4(5·0) *versus* 12·0(6·4) days in the control group; *P* = 0·002) (*Table* *5*), with no difference in LOS on meta‐analysis (data not shown).

Burden and colleagues^40^ (median percentage weight loss 4·1 (i.q.r. 1·7–7·0) in the intervention group *versus* 6·7 (2·6–10·8) in the control group; *P* = 0·016) and Smedley *et al*.^33^ (less weight loss in group 2, *P* = 0·05) were able to demonstrate a reduction in preoperative weight loss with their interventions that was not seen in other studies^31^, ^32^, ^41^. No differences in functional walking ability^30^ or psychological outcomes^30^, ^32^, ^33^, ^41^ were found (*Table* *5*).

#### Psychological studies

Chaudhri and co‐workers^34^ reported a reduction in LOS in the intervention group (8 *versus* 10 days in the control group; *P* = 0·029), which was attributed to fewer delayed discharges owing to stoma proficiency (*Table* *6*). Haase *et al*.^35^ found no difference in overall complications between either of their interventions and the control. Neither psychological intervention had any effect on the measured psychological outcomes^34^, ^35^ (*Table* *6*).

**Table 6:** Summary of outcomes and results for psychological prehabilitation

Reference	Primary study outcome	Postoperative outcomes*	Functional outcomes*	Psychological outcomes*
**Chaudhri *et al*.** ^34^	Time to stoma proficiency, LOS	LOS: 8 *versus* 10 days, *P* = 0·029		HADS postop.: anxiety 33% *versus* 32%; depression 17% *versus* 24%
**Haase *et al*.** ^35^	Systemic analgesic consumption via PCA			EORTC‐QLQ and GIQLI: *P* n.s.
Group 1		Wound infection: 3 of 20 *versus* 3 of 18 Delirium: 0 of 20 *versus* 0 of 18 LOS: overall median (range) 12·5 (11–14) days		
Group 2		Wound infection: 4 of 22 *versus* 3 of 18 Delirium: 1 of 22 *versus* 0 of 18 LOS: median (range) 12·5 (11–14) days		

* Comparative data show intervention and control results respectively. LOS, length of hospital stay; HADS, Hospital Anxiety and Depression Scale; PCA, patient‐controlled analgesia; EORTC QLQ, European Organisation for Research and Treatment of Cancer Quality of Life Questionnaire; GIQLI, GastroIntestinal Quality of Life Index.

#### Comprehensive geriatric assessment with optimization

McDonald and colleagues^54^ demonstrated a reduction in the mean number of complications per patient with the intervention (0·9 *versus* 1·4 in the control group, 95 per cent c.i. −0·13 to −0·89; *P* < 0·001), despite a significantly higher incidence of delirium in the intervention group (52 of 183 *versus* 8 of 143, 95 per cent c.i. 3·06 to 14·65; *P* < 0·001) (*Table* *7*).

Two studies demonstrated a significant reduction in LOS with intervention: median 4 *versus* 6 days respectively (95 per cent c.i. −1·06 to −4·21; *P* < 0·001) in the study by McDonald *et al*.^54^, and a median of 7 (range 5–12) *versus* 9 (7–14) days respectively (*P* = 0·001) in that by Indrakusuma and colleagues^55^. McDonald and co‐workers^54^ demonstrated an improvement in independence on discharge with the intervention (114 of 183 *versus* 73 of 143 respectively, 95 per cent c.i. 1·02 to 2·47; *P* = 0·04). Hempenius *et al*.^37^ observed an improvement in psychological outcome with intervention (SF‐36® bodily pain scores were the same or better in 57 of 127 *versus* 41 of 133 in the control group; OR 0·49, 95 per cent c.i. 0·29 to 0·82) (*Table* *7*).

#### Smoking studies

The smoking cessation trial^39^ did not find a reduction in either complications or LOS with intervention (*Table* *8*).

**Table 7:** Summary of outcomes and results for comprehensive geriatric assessment with optimization prehabilitation

Reference	Primary study outcome	Postoperative outcomes*	Functional outcomes*	Psychological outcomes*
Hempenius *et al*.^37^	Postop. delirium	Complications (> 1): 42 of 127 *versus* 38 of 133 (OR 1·24 (95% c.i. 0·73, 2·10)) Pulmonary: 31 of 127 *versus* 27 of 133 Wound: 13 *versus* 12, *P* = 0·37 Delirium: 12 of 127 *versus* 19 of 133 (OR 0·63 (0·29, 1·35)) LOS: 8 *versus* 8 days	Independence on discharge: 76 of 127 *versus* 87 of 133 (OR 1·84 (1·01, 3·37))	SF‐36® bodily pain same or better: 57 of 127 *versus* 41 of 133 (OR 0·49 (0·29, 0·82))
Indrakusuma *et al*.^55^	30‐day mortality, delirium, LOS	Pneumonia: 37 of 221 *versus* 31 of 222 Wound: 18 of 221 *versus* 26 of 222 Delirium: 22 of 221 *versus* 27 of 222 LOS: 7 (range 5–12) *versus* 9 (7–14) days; *P* = 0·001		
McDonald *et al*.^54^	LOS, readmissions and level of care at discharge	Complications: mean 0·9 *versus* 1·4 (95% c.i. −0·13, −0·89), *P* < 0·001 Delirium: 52 of 183 *versus* 8 of 143 (95% c.i. 3·06, 14·65), *P* < 0·001 Pulmonary: 18 of 183 *versus* 25 of 143 Wound: 4 of 183 *versus* 8 of 143 LOS: median 4 *versus* 6 days (95% c.i. −1·06, −4·21), *P* < 0·001	Discharge home with self‐care: 114 of 183 *versus* 73 of 143 (95% c.i. 1·02, 2·47), *P* = 0·04	
Ommundsen *et al*.^38^	Complications, CDC grade ≥ II	Any complication: 40 of 52 *versus* 55 of 62 CDC grade ≥ II: 36 of 52 *versus* 47 of 62 LOS: 8 *versus* 8 days	Discharged directly home: 38 of 57 *versus* 38 of 65, *P* = 0·2	

* Comparative data show intervention and control results respectively. OR, odds ratio; LOS, length of hospital stay; SF‐36®, Short Form 36; CDC, Clavien–Dindo classification.

**Table 8:** Summary of outcomes and results for smoking cessation prehabilitation

Reference	Primary study outcome	Postoperative outcomes*	Functional outcomes*	Psychological outcomes*
Sørensen and Jørgensen^39^	Postop. wound and tissue complications within 30 days	Any complication: 11 of 27 *versus* 13 of 30 Pneumonia: 3 of 27 *versus* 4 of 30 Wound: 3 of 27 *versus* 4 of 30 LOS: median (i.q.r.) 11 (10–13) *versus* 11 (8–14) days		

* Comparative data show intervention and control results respectively. LOS, length of hospital stay.

## Discussion

This systematic review has found evidence from a number of trials that exercise, multimodal, nutrition and CGA with optimization prehabilitation programmes may reduce the number of postoperative complications after elective surgery for gastrointestinal and urological cancers. It has shown evidence that multimodal, nutritional, psychological and CGA interventions (but not exercise interventions or smoking cessation alone) may reduce LOS. In particular, the small number of studies that selected high‐risk, frail or older patients were more likely to report improvements in either complications or LOS compared with studies that included all patients. Equally, studies conducted in patients undergoing oesophageal and upper gastrointestinal surgery, known to be associated with high levels of postoperative morbidity and mortality, were more likely to demonstrate reductions in pulmonary complications. However, conclusions are limited by the methodological quality of included studies, in particular the lack of blinding of participants in all except three studies. Significant heterogeneity of interventions also limits comparison. Adherence to exercise, multimodal and nutritional interventions was generally high; however, it is possible that participant selection bias and lack of blinding may have resulted in more motivated patients being recruited.

National and international guidelines^57^, ^58^, ^59^ recommend that CGA should be performed in all patients over the age of 70 years with a diagnosis of cancer to try to predict treatment toxicity and postoperative complications, and to aid in shared decision‐making. However, there remain very few studies of CGA in surgical cancer populations, and the majority of these are limited to its role in risk prediction and prognostication^60^, ^61^. This systematic review identified only two RCTs^37^, ^38^ evaluating CGA and tailored interventions. It is worth noting that the median age of patients in studies included in this review was only 68 years, with patients in the exercise‐alone interventions having a median age of only 63 years. Only seven of the 33 studies in this review had a median age greater than 75 years. This suggests that many prehabilitation studies to date either failed to recruit older patients due to the location or nature of the interventions or they excluded older patients owing to a perceived risk of the interventions, despite mounting evidence^62^, ^63^ that exercise‐based interventions are safe in older individuals.

This review also demonstrated that improvements in preoperative functional measures can be made with exercise prehabilitation (measured by CPET), multimodal interventions (measured using 6MWT) and nutritional prehabilitation (reduction in preoperative weight loss). However, the link between small statistically significant improvements in these variables and clinical outcomes is not clear.

A number of previous systematic reviews have examined individual components of prehabilitation in varying surgical populations: exercise^18^, ^19^, ^20^, ^64^, ^65^, exercise in frail individuals^16^, multimodal interventions^13^, ^14^, ^15^, multimodal interventions in frail individuals^12^, nutrition with and without exercise^66^, and psychological interventions^11^. All of these, including the present review, have been limited by the quality of the underlying evidence. This is the first review that included all modalities of prehabilitation of relevance to the older adult.

Prehabilitation programmes, regardless of the individual components they comprise, are complex multicomponent interventions, and thus should be evaluated as such. The Medical Research Council in the UK has published a clear framework for evaluating and conducting trials involving complex interventions^67^. Two of the potential reasons for negative findings in prehabilitation studies are either that the interventions are too standardized to enable reproducible delivery or that, in efforts to provide truly personalized programmes, no two individuals receive the same intervention. Equally, although there is accumulating evidence that multimodal prehabilitation is likely to be more beneficial than using a single modality, future trials that use methodologies designed for evaluating complex interventions will be able to determine which components are most beneficial for different patients and why.

This review is limited by the heterogeneity of outcomes reported. LOS and complications were selected as primary outcomes for this review; however, a number of studies were powered to detect changes in other primary outcomes and therefore may have been inadequately powered for the primary outcomes of this review. The majority of trials in prehabilitation are relatively small, and this may contribute towards reporting bias of trials with statistically significant outcomes. Heterogeneity of studies may have also contributed to some analyses attaining statistical significance inappropriately. The wide date range of included studies may have added to the heterogeneity, as perioperative care has evolved over the past 20 years with the introduction of enhanced recovery pathways and laparoscopic surgery. Another potential limitation is that diverse surgical procedures with a range of complication rates have been compared. This may have resulted in some analyses not reaching significance, and will have contributed towards heterogeneity on meta‐analysis. For the purpose of this review, a large number of studies were excluded at full‐text review due to lack of reporting of LOS or complications, which are considered core outcomes for surgical trials^68^, ^69^. In particular, a number of trials of psychological interventions^70^, ^71^, ^72^, ^73^, ^74^, ^75^ were excluded for this reason. Of note, only one preoperative smoking cessation trial^39^ and no studies in gynaecological cancer surgery met the inclusion criteria. The main strength of this review is the comprehensive nature, whereby all current prehabilitation modalities in abdominal cancer surgery were included. This means that the review is of relevance to a wide range of surgical specialties, identifies gaps in the current evidence base, and will be of interest to commissioners looking to fund prehabilitation services.

The reporting of outcomes presented a challenge in this review owing to the range of outcome measures used; this reflects complex interventions and the inability to compare them directly, and raises an important issue for researchers. The evidence base for prehabilitation might be stronger if a core outcome set could be used in all trials, irrespective of modality of prehabilitation or surgical population, to facilitate comparison of interventions. The StEP‐COMPAC group (Standardising Endpoints in Perioperative Medicine) have already made progress in this regard in perioperative medicine^76^, ^77^, ^78^, ^79^. Initiatives such as the DiSCO (Defining Standards in Colorectal Optimisation) project led by researchers in the West of Scotland, which aims to create key sets of standards for prehabilitation in collaboration with patients, their caregivers and the public, will be vital in ensuring that results are relevant to service users as well as clinicians, and to the successful promotion of patient‐centred care. Future studies also need to evaluate strategies for implementation and the associated costs to enable adequate investment at a time of increasing healthcare costs.

## Supporting information


**Appendix** **S1** Search strategy
**Table S1** General characteristics of the included studies
