# Supplementary material for: Prehabilitation in elective abdominal cancer surgery in older patients: systematic review and meta‐analysis
Source: BJS Open. 2020 Sep 22;4(6):1022–41. doi: 10.1002/bjs5.50347 (PMC7709363; doi:10.1002/bjs5.50347)
Supplement: Supplementary file 1 — Appendix S1 Search strategy Table S1 General characteristics of the included studies [file BJS5-4-1022-s001.docx]

**BJS5_50347**

**Prehabilitation in elective abdominal cancer surgery in older patients: systematic review and meta-analysis**

**S. L. Daniels, M. J. Lee, J. George, K. Kerr, S. Moug, T. R. Wilson, S. R. Brown and L. Wyld**

**Appendix S1** Search strategy

MEDLINE search

1. Perioperative.mp
2. peri-operative.mp
3. pre-operative.mp
4. preoperative care/exp
5. pre-hab*.mp
6. prehab*.mp
7. pre hab*mp
8. 1 OR 2 OR 3 OR 4 OR 5 OR 6 OR 7
9. exercise/exp
10. physiotherapy.mp
11. preconditioning.mp
12. exercise therapy/exp
13. circuit-based exercise/exp
14. 9 OR 10 OR 11 OR 12 OR 13
15. nutrition therapy/exp
16. diet therapy.mp
17. nutrition assessment/exp
18. dietician assessment
19. 15 OR 16 OR 17 OR 18
20. smoking cess*.mp
21. nicotine replacement.mp
22. 20 OR 21
23. alcohol cessation.mp
24. alcohol reduction.mp
25. 23 OR 24
26. comprehensive geriatric assessment.mp
27. geriatric optimi*.mp
28. geriatric intervention.mp
29. 26 OR 27 OR 28
30. psychological*.mp
31. cognitive therap*.mp
32. psychotherapy*.mp
33. 30 OR 31 OR 32
34. abdominal surgery.mp
35. gastrointestinal surgery/exp
36. Digestive system Surgical Procedures/exp
37. gynecological surgical procedures/exp OR gynaecological surgery.mp
38. urological surgical procedures/exp OR urological surgery.mp
39. gastrointestinal neoplasms/exp
40. oncolog*.mp
41. malign*.mp
42. 34 OR 35 OR 36 OR 37 OR 38 OR 39 OR 40 OR 41
43. 14 OR 19 OR 22 OR 25 OR 29 OR 33
44. 8 AND 42 AND 43

**Table S1** General characteristics of the included studies

a. Exercise

| Study | Study design and location | Population | Study groups | Sample size | Age in years (control) |
| --- | --- | --- | --- | --- | --- |
| Banerjee 2018 | Feasibility RCT, single centre, UK | Bladder cancer including neoadjuvant | Supervised, individual, high-intensity, aerobic interval training on cycle ergometer; 30 minutes per session, twice weekly for 3-6 weeks. Compared to standard care. | Intervention 30; control 30 | Mean 71.6 (72.5) |
| Barberan-Garcia 2018 | RCT, single centre, Spain | Abdominal surgery (75% oncological)  Age >70 ± ASA 3/4 and Duke Activity Status index score ≤46 | Supervised, personalised high-intensity endurance exercise programme plus programme to promote physical activity. Home-based, physiotherapist led. 45 minutes 1-3 times a week, 6 weeks. Compared to standard care. | Intervention 62, control 63 | Mean 71 (71) |
| Boden 2018 | RCT, multi-centre Australasia | Upper GI surgery (68% oncological) | Supervised in hospital pulmonary physiotherapy education and training session for 30 mins plus educational booklet. Compared to educational booklet. | Intervention 218, control 214 | Median 63.4 (67.5) |
| Carli 2010 | RCT, single, Canada | Colorectal surgery (62% oncological) | Home-based, aerobic (cycle) and strengthening programme based on % maximal heart rate. 20-45 minute sessions daily for 4 weeks. Compared to advice to walk more and breathing exercises. | Intervention 58, control 54 | Mean 61(50) |
| Dronkers 2010 | Feasibility RCT, single centre Netherlands | Colon cancer  Age >60 years | Supervised resistance, moderate intensity aerobic exercise, inspiratory muscle training and training in functional activities in hospital twice a week for 60 minutes for 2-4 weeks plus advice to walk or cycle for 30 minutes a day. Compared to advice only. | Intervention 22, control 20 | Mean 71.1 (68.8) |
| Dunne 2016 | RCT, single centre, UK | Colorectal liver metastases | Supervised, high-intensity cycle, interval training programme, 45 minutes, three times a week for 4 weeks. Compared to standard care. | Intervention 20, control 18 | Median 61 (62) |
| Santa Mina 2018 | Feasibility RCT, multi-centre Canada | Prostate cancer | Home-based, unsupervised, moderate intensity exercise for 60 minutes 3-4 times a week plus daily pelvic floor muscle exercises (including booklet). Compared to booklet plus pelvic floor exercises only. | Intervention 44, control 42 | Mean 61.2 (62.2) |
| Soares 2013 | RCT, multi-centre, Brazil | Upper GI surgery (78% oncological), open surgery only | Supervised, hospital-based physical therapy sessions including aerobic, stretching and respiratory muscle training for 50 minutes twice a week for 2-3 weeks plus daily self-directed respiratory exercises. Compared to standard care. | Intervention 16, control 16 | Median 58.5 (55) |
| Yamana 2015 | RCT, single centre Japan | Oesophageal cancer, including neoadjuvant | Supervised pulmonary rehabilitation programme including aerobic exercises on bike in hospital for 60 minutes, daily, for at least 7 days. Compared to standard care. | Intervention 30, control 30 | Mean 68.33 (65.9) |

b. Multi-modal

| Study | Study design and location | Population | Study groups | Sample size | Age in years (control) |
| --- | --- | --- | --- | --- | --- |
| Bousquet-Dion 2018 | RCT, single centre, Canada | Colorectal cancer | Home and hospital based aerobic and resistance exercise, Dietician assessment and prescription of supplements to achieve 1.2g protein/day, 60-minute psychological consultation (anxiety). Exercise 30-45 minutes/ session, 3-4 times a week for 4 weeks. Compared to post-op rehabilitation. | Intervention 37, control 26 | Median 74 (71) |
| Chia 2016 | Prospective cohort, single centre, Singapore | Colorectal cancer, >65 years, frail | Education, cardiovascular strengthening, attention to nutrition, post-operative rehabilitation. Twice per week, duration not stated. Compared to historical control. | Intervention 57, control 60 | Median 79 (81) |
| Gillis 2014 | RCT, single centre, Canada | Colorectal cancer | Home-based aerobic and resistance training, dietician assessment and prescription of protein supplementation (1.2g protein/kg), psychological assessment and DVD. 40 minutes/ session, 3 times a week for 4 weeks. Compared to post-operative rehabilitation. | Intervention 38, control 39 | Mean 66 (66) |
| Jensen 2016 | RCT, single centre, Netherlands | Bladder cancer | Strengthening and endurance (supervised instruction session then self-directed) using step trainer. 30-60 minutes daily for 2 weeks. Included post-operative rehabilitation. Nutritional screening and supplementation and lifestyle advice on smoking and alcohol were part of standard care. Compared to standard care. | Intervention 50, control 57 | Mean 69 (71) |
| Kaibori 2013 | RCT, single centre, Japan | Hepatocellular carcinoma with chronic liver injury Childs Pugh A or B | Personalised aerobic exercise programme plus specific diet recommended for liver disease. 60 minutes per session, 3 times a week for 4 weeks. Compared to diet recommendation alone. | Intervention 26, control 25 | Mean 68 (71) |
| Li 2013 | Prospective, non-randomised, single centre, Canada | Colorectal cancer | Personalised programme of home-based, moderate aerobic exercise plus resistance for 30 minutes per session, three times a week, dietician assessment, protein supplementation 1.2g/kg/day and psychological advice. Compared to historical control. | Intervention 42, control 45 | Mean 67 (66) |
| Mazzola 2017 | Prospective with historical control, single centre, Italy | Upper GI and pancreatic malignancies, neoadjuvant, frail patients mFI ≥ 2 | Encouraged to exercise by moderate intensity walking for 30 minutes, three times per week. Respiratory exercises using incentive exerciser. Malnourished patients received ONS for 2 weeks prior to surgery, non-malnourished patients for 5-7 days. Compared to historical control | Intervention 41, control 35 | Mean 75 (75) |
| Minnella 2018 | RCT, single centre, Canada | Oesophageal cancer including neoadjuvant | Individualised home-based moderate intensity aerobic and resistance exercise for 30 minutes 3 times a week for 5 weeks plus strengthening exercises for 30 minutes once a week, dietician assessment and prescription of whey ONS 1.2g/kg ideal body weight. Compared to standard care. | Intervention 26, control 25 | Mean 67 (68) |
| Nakajima 2018 | Prospective cohort historical control, single centre Japan | HPB malignancies, open surgery only, excluding neoadjuvant | Home-based unsupervised moderate aerobic exercise and resistance training for 60 minutes, three times a week. Nutritional therapy involved taking an amino acid supplement after exercise. Compared to historical control (propensity matched) | Intervention 76, control 76 | Median 69 (69) |
| Souwer 2018 | Prospective cohort with historical controls, single centre, Netherlands | Colorectal cancer ≥ 75 years, including neoadjuvant | Supervised aerobic and resistance exercise 30-45minutes per session twice a week for 4-6 weeks, geriatric screening and intervention, dietician assessment and prescription of protein supplementation, Colorectal nurse specialist psychosocial support, referral for cardiac or pulmonary optimisation if indicated. Compared to historical control. | Intervention 86, control 63 | Median 81 (81) |

c. Nutrition

| Study | Study design and location | Population | Study groups | Sample size | Age in years (control) |
| --- | --- | --- | --- | --- | --- |
| Burden 2017 | RCT, multi-centre UK | Colorectal cancer, malnourished | ≥10day preoperative ONS 400ml/d between meals plus dietary advice leaflet to increase energy and protein. Compared to dietary advice alone | Intervention 54, control 62 | Mean 65 (65) |
| Gillis 2016 | RCT, single centre, Canada | Colorectal cancer | 4 weeks preoperative individualised ONS prescription 1.2g/kg protein/day plus dietary counselling. Compared to dietary counselling plus placebo. | Intervention 22, control 21 | Mean 68 (69) |
| Kabata 2015 | RCT, single centre, Poland | GI and abdominal cancers, non-malnourished | ≥14 days preoperative ONS 400ml/day. Compared to standard care | Intervention 54, control 48 | Median 60 (67) |
| Kong 2018 | RCT, single centre, Korea | Gastric cancer, malnourished | 14 days preoperative ONS 500kcal/day. Compared to standard care | Intervention 65, control 62 | Mean 61.9 (62.3) |
| Macfie 2000 | RCT, single centre, UK | Major GI surgery (‘majority’ oncological) | Group 1: ≥10 days preoperative ONS 400ml/day  Group 2: ≥10 days preoperative ONS 400ml/day plus 7 days post-operative ONS  Compared to standard care | Intervention 1 24  Intervention 2 24, control 25 | Mean 62 (64)  63 (64) |
| Manasek  2016 | Prospective cohort, multicentre, Czech Repub. | Colorectal cancer, including neoadjuvant | ≥10 days preoperative ONS 400ml/day between meals plus dietary advice. Compared to dietary advice alone | Intervention 52, control 105 | Mean 64 |
| Smedley 2004 | RCT, multi-centre, UK | Colorectal surgery (62% oncological) | Group 1: ≥7 days preoperative ONS – advised to take small frequent doses between meals.  Group 2: ≥7 days preoperative ONS – advised to take small frequent doses between meals plus post-operative ONS  Compared to standard care | Intervention 1 41  Intervention 2 32, control 44 | Mean 61 (63)  Mean 55 (63) |

d. Psychological

| Study | Study design and location | Population | Study groups | Sample size | Age in years (control) |
| --- | --- | --- | --- | --- | --- |
| Chaudhri 2005 | RCT, single centre, UK | Colorectal requiring stoma >69% oncological | Community stoma education; two 45-minute home visits preoperative with a community colorectal nurse specialist. Compared to standard care. | Intervention 21, control 21 | Median 69 (62) |
| Haase 2005 | RCT, single centre, Germany | Colorectal cancer | Group 1: Guided imagery audio recording with music to be played three times a day 2 days preoperatively and for 30 days post-op  Group 2: Relaxation audio recording with music to be played three times a day 2 days preoperatively and for 30 days post-operatively. Compared to standard care. | Intervention 1 20,  intervention 2 22, control 18 | Mean 65 (66) |

e. Comprehensive Geriatric Assessment with Optimisation

| Study | Study design and location | Population | Study groups | Sample size | Age in years (control) |
| --- | --- | --- | --- | --- | --- |
| Hempenius 2013 | RCT, multi-centre, Netherlands | Abdominal, GI, breast, ENT, lung cancers (52% major surgery)  >65 years, frail GFI >3 | CGA, best supportive care and prevention of delirium post-operative by geriatrician led team. Including medication optimisation, co-morbidity review, nutrition, visual/hearing loss, mobility, depression, preventative pharmacological measures. One pre-operative consultation plus daily post-operative geriatric nurse reviews.  Compared to standard care. | Intervention 127, control 133 | Mean 77 (78) |
| Indrakusuma 2015 | Retrospective single centre Netherlands | Colorectal cancer, >70 years | CGA and medical optimisation, nursing interventions, blood transfusion and nutritional supplementation by geriatric specialists. Compared to historic control. | Intervention 221, control 222 | Median 77 (77) |
| McDonald 2018 | Case control study, single centre, USA | Major abdominal surgery, >65 years with risk factors or >85 without | CGA and optimisation of medications, nutrition, cognition, advanced care planning and risk reducing strategies by geriatric led team plus daily post-operative review.  Compared to pre-intervention standard care. | Intervention 183, control 143 | Mean 76 (72) |
| Ommundsen 2017 | RCT, multi-centre, Norway | Colorectal cancer, >65 years, frail VES plus clinical criteria | CGA and optimisation of medications, dietary advice, vitamin and iron supplementation by geriatric doctor. Plus, post-operative physiotherapy for COPD.  Compared to standard care. | Intervention 57, control 65 | Mean 78 (79) |

f. Smoking cessation

| Study | Study design and location | Population | Study groups | Sample size | Age in years intervention (control) |
| --- | --- | --- | --- | --- | --- |
| Sorensen 2003 | RCT, single centre, Denmark | Colorectal disease (70% oncological) | Counselling and pharmacotherapy with nicotine replacement therapy, one visit plus telephone call. Compared to standard care. | Intervention 30, control 27 | Median 65 (66) |

*Abbreviations*

*ASA; American Society of Anesthesiologists, mFI; Modified Frailty Index, ONS; oral Nutritional Supplement; HPB; Hepatobiliary, CGA; Comprehensive Geriatric Assessment, VES; Vulnerable Elders Survey, GFI; Groningham Frailty Index.*
